# Control-theoretic integration of stimulation and electrophysiology for cognitive enhancement

**DOI:** 10.3389/fnimg.2022.982288

**Published:** 2022-11-18

**Authors:** Matthew F. Singh, Michael W. Cole, Todd S. Braver, ShiNung Ching

**Affiliations:** ^1^Electrical and Systems Engineering, Washington University in St. Louis, St. Louis, MO, United States; ^2^Center for Molecular and Behavioral Neuroscience, Rutgers University, Newark, NJ, United States; ^3^Psychological and Brain Science, Washington University in St. Louis, St. Louis, MO, United States

**Keywords:** neurostimulation, transcranial electric stimulation, control theory and control engineering, cognitive enhancement, brain dynamics

## Abstract

Transcranial electrical stimulation (tES) technology and neuroimaging are increasingly coupled in basic and applied science. This synergy has enabled individualized tES therapy and facilitated causal inferences in functional neuroimaging. However, traditional tES paradigms have been stymied by relatively small changes in neural activity and high inter-subject variability in cognitive effects. In this perspective, we propose a tES framework to treat these issues which is grounded in dynamical systems and control theory. The proposed paradigm involves a tight coupling of tES and neuroimaging in which M/EEG is used to parameterize generative brain models as well as control tES delivery in a hybrid closed-loop fashion. We also present a novel quantitative framework for cognitive enhancement driven by a new computational objective: shaping how the brain reacts to potential “inputs” (e.g., task contexts) rather than enforcing a fixed pattern of brain activity.

## 1. Introduction

Non-invasive neurostimulation techniques are more frequently being combined with neuroimaging (e.g., Bergmann et al., 2012; Garcia-Cossio et al., 2016; Reinhart and Nguyen, 2019), as an effective way to integrate perturbation and monitoring approaches in cognitive neuroscience studies. The term non-invasive neurostimulation refers to a range of techniques, including transcranial magnetic stimulation (TMS), transcranial electrical stimuluation (tES), and transcranial focused ultrasound (tFUS). In this perspective, we focus primarily on tES and how it might be integrated with high temporal-resolution neuroimaging techniques such as magnetoencephalography (MEG) and electroencephalography (EEG).

Transcranial electrical stimulation (tES) involves the use of stimulating scalp electrodes to generate small electric fields throughout the head (Paulus, 2011). The strength of tES is typically described in terms of electric current (mAs) for experimental purposes, while the induced neuronal effects are typically reported as voltage gradients (e.g., Antonenko et al., 2021). Unlike transcranial magnetic stimulation (TMS), tES induces relatively small changes in neuronal membrane potential, but is potentially more versatile due to its ease of construction, small profile (e.g., portability), more mild side-effects, and support for a large number of channels. Despite this promise, several lines of evidence indicate that neurostimulation has not yet reached its full potential, including issues of high inter-subject variability (López-Alonso et al., 2014) and inconsistent/weak effects (Horvath et al., 2015a,b).

tES protocols are broadly grouped into those that employ a constant, DC voltage (tDCS) and those that involve some form of alternating current (tACS), which need not be periodic. Although the majority of existing literature concerns tDCS (which was the earliest form of tES), increasing emphasis is being placed upon the potential of dynamic stimulation (i.e., tACS). Here, we discuss the potential for deriving (near)-optimal stimulation protocols which, depending upon the objective, may be constant (DC), periodic, or neither. We also note the existence of transcranial random noise stimulation (tRNS) in which high-frequency random electric currents are used (Terney et al., 2008).

In this tutorial, we describe how formal analyses of control-theory can be used to optimize neurostimulation designs and to develop new stimulation objectives. Control theory is a branch of engineering that treats the problem of producing desired behavior in (“controlling”) dynamical systems by manipulating inputs to the system. The methods and objectives of control theory are broad, and we do not seek to provide a self-contained introduction to control (interested readers should see, e.g., Kirk, 2004; Schiff, 2010). Rather, we present this tutorial with two goals.

1. To describe control-theoretic concepts that can inform the design and implementation of neuroimaging+tES experiments (both heuristically and formally).
2. To advocate for control-theoretic measures (reachability) as a means to bridge between microscale neural computation and macroscale tES.

Both goals leverage a tight integration of neuroimaging and tES. Neuroimaging data is essential for identifying models of underlying brain activity (see Section 7.2) and for monitoring ongoing brain activity to guide tES delivery. We particularly emphasize that the state of a non-linear system (like the brain) determines its response to additional inputs (Section 4) and knowing this state is essential for achieving the desired downstream effects. Previous studies have demonstrated how online monitoring of brain activity (*via* neuroimaging) benefits neurostimulation design and delivery (Bergmann et al., 2012; Reinhart and Nguyen, 2019).

We first present some fundamental insights from control theory and, using case-problems, illustrate the potential of such approaches to advance neurostimulation. We then present control-theory as an integrative perspective for understanding the neural processes that give rise to adaptive behavior. This perspective leads to a new formulation of neurostimulation objectives as control-theoretic (reachability) conditions to enhance cognitive control. Our objectives are particularly relevant for cognitive enhancement of individuals who have not suffered additional brain injury or cognitive decline, including healthy subjects, as it does require identifying specific etiology.

## 2. Control principles

A central insight of control theory is that, under suitable conditions, spatial and temporal degrees of freedom may be interchanged so that a system with more internal states than inputs may still be controllable *via* the temporal structure of inputs. The auditory system is a prime example of this phenomenon in neuroscience. A single actuator to the cochlea (movement of the stapes) controls thousands of spatial degrees of freedom (hair cells). This control is realized by internal dynamics of the basilar membrane whose varying thickness produces a spatial gradient of mechanical resonances (often compared to a Fourier decomposition). Thus, asymmetric dynamics enable a conversion from temporal to spatial degrees of freedom (or vice-versa). This notion is formalized *via* Lie Algebras in geometric control theory (Brockett, 1973).

In a dynamical system, the future state (e.g., spatial activity pattern) of a system is a function of its current state plus any additional inputs:


(1) $$\begin{array}{l} ẋ=f\left(x_{t}\right)+Bu_{t} \end{array}$$

Here, *x* is an *n*-element vector of brain state-variables (e.g., *x*_*i*_ is the activity of the *i*^*th*^ population) and *f* is the dynamical systems model. The *k*-element vector *u*_*t*_ describes the applied current (each element representing an independent stimulation channel) and the *n*×*k* matrix *B* contains the relative gains between stimulation channels and brain state-variables, analogous to a lead-field matrix. In this setup, we treat the instantaneous impact of tES as additive (i.e., an additional current source to Ohmic models). This assumption is justified when *f* is a detailed biophysical neuron model, but future study is needed to test whether the same assumptions hold for reduced population-level models. For instance, at population level, the modulation of voltage-gated channels might be better approximated by a change in connection strength rather than direct modulation of population-level activity. However, we use this formulation, at present, as it is the most common, parsimonious, and accessible.

**Figure 1:**
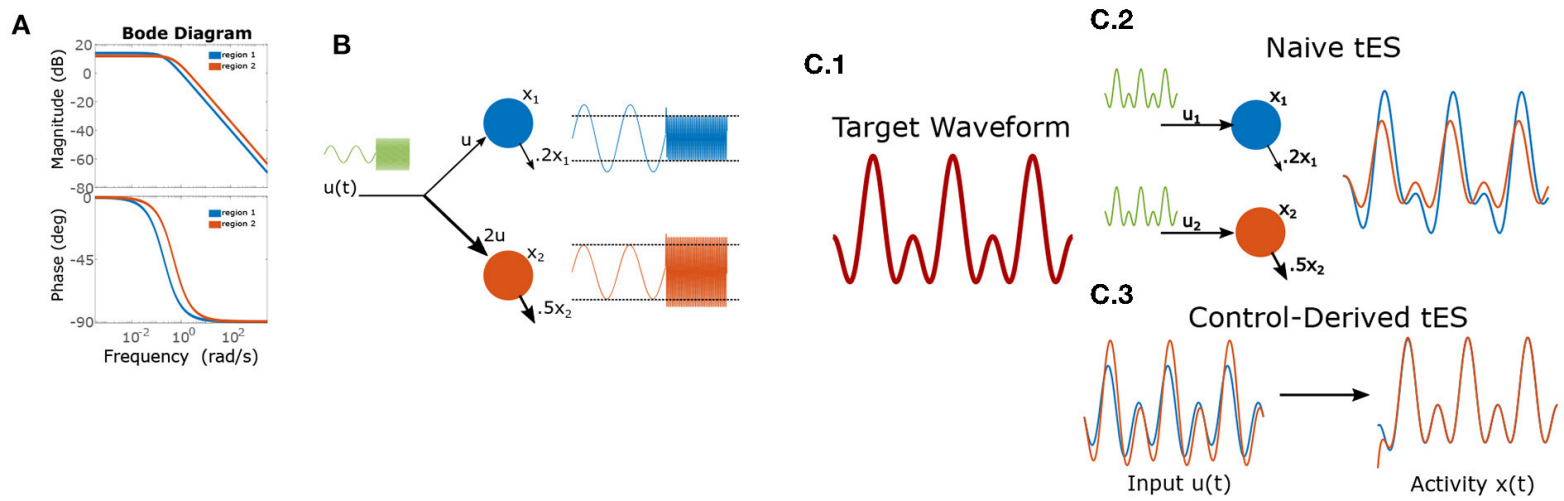
Frequency dependent gains and phase-shifts for a pair of linear leaky-integrators. **(A)** Bode-plot of gains (top) and phase-shifts (bottom). **(B)** Heterogeneous dynamics enable reversal of relative amplitudes for two integrators (“brain regions”) based upon input frequency. The left-half of each timeseries shows a low-frequency input which is magnified by region 1, while the right-half shows the effect of a high-frequency input which results in greater power for region 2). **(C)** Using control to induce a target wave in two brain areas. **(C.1)** The target is composed of two sinusoidal components. **(C.2)** Applying the same wave to each node results in desynchronization and temporal distortion. **(C.3)** Control theory identifies (slightly) asynchronous inputs to produce the desired (synchronous) output behavior.

The internal dynamics of this system alter multiple aspects of how the system responds to an input signal (*u*_*t*_). For example, in a linear system with periodic forcing, these effects may be fully decomposed into two components: frequency-dependent amplification and frequency-dependent phase-shifts. These properties are depicted graphically as a “Bode plot” (e.g., Figure 1A) and a recent study empirically estimated these functions for neurostimulation of macaques (Yang et al., 2021). Frequencies with the highest gain are referred to as “resonant”. In humans, a study by Reinhart and Nguyen (2019) demonstrated the heuristic power of applying tACS at frequencies likely to be near-resonant (those dominant at rest). Component-wise differences in these properties (e.g., regional heterogeneity; Demirtas et al., 2019; Wang et al., 2019; Singh et al., 2020) can be exploited for control. We expound upon these implications in two illustrative cases.

## 3. Control concepts I: Time-domain

Throughout this section, we will use cartoon models of brain dynamics to demonstrate underlying ideas while retaining analytic, visually-compact solutions. Unlike the non-linear brain, all (stable) linear dynamical systems can be reduced to the action of convolving the input timeseries with a system-specific kernel. For example, the simple integrator system below (*x*(*t*)) acts equivalently to convolving inputs (*u*(*t*)) with *be*^−*at*^:


(2) $$\begin{array}{l} ẋ=-ax+bu\left(t\right) \end{array}$$

with ẋ denoting the temporal derivative (the instantaneous rate of change in *x* per unit time). For oscillating inputs (i.e., tACS) linear systems act as filters amplifying/attenuating certain frequencies and generating frequency-specific phase-shifts. For pure sinusoidal input (0-phase) and a frequency of ω in terms of radians (*u*(*t*) = *sin*(ω*t*)), this system has the analytic solution:


(3) $$\begin{array}{l} x\left(t\right)=c\left(\omega ,t\right)+\frac{b}{\sqrt{\omega^{2}+a^{2}}}sin\left(\omega t-\phi \left(\omega \right)\right) \end{array}$$

with phase shift: ϕ = *tan*^−1^(ω/*a*) and transient exponential component: *c*(ω, *t*) = (*x*_0_+*b*sin(ϕ))*e*^−*at*^. The frequency dependent gain is thus inversely proportional to $\sqrt{\omega^{2}+a^{2}}$. Thus, both the phase and amplitude of induced oscillations (e.g., *via* tACS) depend upon the input frequency (ω) and the local dynamics (*a*). We explore these properties in two empirically-relevant case problems using the simple integrator model for pedagogy (more realistic, non-linear models may be necessary for application and are treated later) and use generic numbers rather than empirical values. In non-linear systems, such characterizations become more nuanced and depend upon the input magnitude and the current state of the system (i.e., ongoing brain activity). However, as we will demonstrate, even the simplest linear systems do not identically reproduce the temporal properties of their inputs. This feature has significant implications for input design and researchers may thus benefit from using formal methods (i.e., control theory) to identify effective neurostimulation protocols. In large, non-linear systems such as the brain, such imperatives are doubly important.

### 3.1. Case 1: Independent amplitude control

Suppose that an experimenter wishes to selectively modulate the relative magnitude of two regions using a single stimulation channel (one anode + one cathode). Thus, at certain times, she desires that power(region 1)>power(region 2) and at other-times vice-versa (e.g., linked to different stages of neural processing). This problem contains two components: how electrodes should be placed and the stimulation waveform. Without loss of generality, we'll suppose that the first region has a slower decay rate (*a*_1_ = 0.2, *a*_2_ = 0.5) and fix *b*_1_ = 1. Using Equation (3), we have that the gain for region 1 (the longer integrator) is always greater than for region 2 when *b*_2_ ≤ *b*_1_, hence achieving greater power in the second region requires *b*_2_>*b*_1_ (e.g., electrodes are placed closer to region 2). For demonstration we choose *b*_2_ = 2. We solve for the maximal difference in gains by setting the derivative equal zero:


(4) $$\begin{array}{l} \frac{d}{d\omega }\left(\frac{1}{\sqrt{\omega^{2}+a_{1}^{2}}}-\frac{b_{2}}{\sqrt{\omega^{2}+a_{2}^{2}}}\right)=0 \end{array}$$

which gives the solution:


(5) $$\begin{array}{l} \omega =\sqrt{\frac{a_{2}^{2}-b_{2}^{2/3}a_{1}^{2}}{b_{2}^{2/3}-1}} \end{array}$$

for maximizing the difference of region 2 gain vs. region 1 gain. By contrast, region 1 has greatest relative gain for very low-frequency inputs due to its longer time constant. Thus, using very low-frequency (or DC) inputs will differentially engage region 1 (see Figure 1B).

### 3.2. Case 2: Synchronization to a target wave

We now consider the task of creating synchronized periodic behavior in two regions using separate inputs to each region (they are sufficiently distal that we ignore electrical conduction between the two sites). Conventionally, researchers have approached this task by delivering synchronous transcranial alternating-current stimulation (tACS) at the two stimulation sites. However, by Equation (3), it is clear that input currents generate a phase-offset (ϕ) in the state-variables. As with gain (see Case 1), the phase-offset is a function of the intrinsic dynamics and the stimulation frequency (Figure 1A). This means that, even for the cartoon model, synchronously applied stimulation does not (generally) result in synchronous responses due to region-specific phase-offsets in the response. For a simple sine-wave, synchronous responses thus require that input phases differ by the difference of phase-offsets so as to cancel this shift. In the more general case of inducing a multi-component wave (the “target”), a different phase offset applies to each frequency-component of tES (i.e., considering each sine-wave separately), since the phase-offsets are a function of frequency. Likewise, the amplitude of each component must also be rescaled from the target to counteract frequency-dependent gains (see Case 1). More generally, for a target of the form:


(6) $$\begin{array}{l} \hat{x}\left(t\right)=\overset{n}{\underset{i}{\sum }}c_{i}sin\left(w_{i}t+v_{i}\right) \end{array}$$

the matching tACS waveform (ignoring the initial transient) is given by


(7) $$\hat{u}(t)=\frac{1}{b}\sum_{i}^{n}c_{i}\sqrt{a^{2}+w_{i}^{2}}sin\left(w_{i}t+v_{i}+tan^{-1}\left[\frac{w_{i}}{a}\right]\right)$$

Thus, in general, the optimal input waveform differs substantially from the induced effect even in simple models as evidenced by the 2-component target-wave in Figure 1C. These distortions differ between regions and, in non-linear systems, are subject to further interactions with ongoing activity. Brain dynamics are thus a critical consideration for ensuring that stimulation protocols achieve the desired neural effects.

## 4. Control concepts II: Non-linear interactions

**Figure 2:**
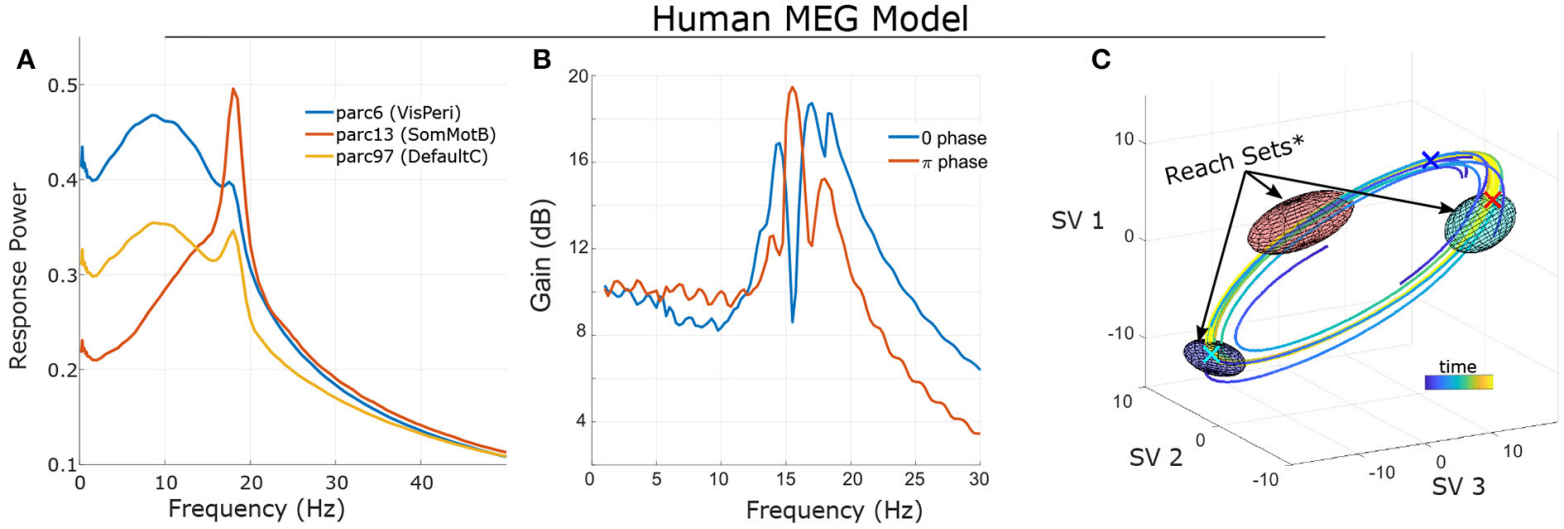
Frequency sensitivity and state-dependence of responses in a single-subject's non-linear model (fit to MEG data; see Singh et al., 2022b. **(A)** Statistical Bode plot showing the average power of responses to simulated tACS input (averaged over many initial conditions, noise realizations). Note the different frequency-sensitivity profiles of illustrated brain regions (regions/networks from the 17-network 100-parcel Schaefer atlas; Schaefer et al., 2017). **(B)** Different initial conditions (phase on a limit cycle) change the response profile of non-linear systems. **(C)** State-space visualization of the limit cycle and some trajectories converging onto it. Control was modeled as two independent stimulation input channels uniformly targeting left Frontoparietal Network and left Default Mode Network (1 degree-of-freedom each). Ellipsoids indicate the reach sets for input after 1/3 period and with *L*_2_ bounds (root-sum-of-squares) ||*u*||_2_ ≤ 4. Initial conditions are marked by “X” in the corresponding color. Note that the size/orientation of reach sets differ based upon initial condition. *For visualization purposes, reach sets are approximated by first-order expansion. See Supplementary material for details.

The general motivation for control extends to non-linear systems as well: dynamics generate temporal asymmetries which can be leveraged for control; note however that non-linear control analysis generally requires numerical methods (simulation and optimization) as opposed to deriving closed-form solutions. These algebraic asymmetries arise due to local regional heterogeneity (e.g., the degree of recurrent connections) and the structured nature of brain connectivity (i.e., connections are not all-to-all). These factors lead to region-specific dynamics which can be exploited for control. As a very basic example, we provide the frequency-dependent gain of simulated sinusoidal tACS applied to a brain model estimated from single-subject MEG data (see Larson-Prior et al., 2013; Singh et al., 2022b) for several brain regions (Figure 2A). Since the models are non-linear, this characterization is purely statistical and represents the average signal power (sum-of-squares) of the response across many noisy simulations. For this specific case, we find that, on average, the greatest change in amplitude results from stimulation in the alpha and/or beta bands (which are dominant at rest) and the specific relationship differs markedly between brain regions. However, this characterization, while useful, does not fully capture the behavior of a non-linear system. A key distinction is that in non-linear systems, it is not possible to separate the system's initial state from the influence of input: long-term effects reflect an interaction between the input signal and ongoing activity.

### 4.1. State/phase dependence

To illustrate this dependence, we consider the same subject's model initialized at different points (phases) along a stable oscillation (a “limit cycle”; Figure 2C). We simulated the model (without noise) starting at each phase and with sinusoidal input. Results (Figure 2B) indicate that the frequency-sensitivity depends strongly upon the initial conditions which, when activity is confined to a limit cycle, can be interpreted as phases. We also note that this sensitivity should not be confused with interference. Interference refers to when separable in-phase signals sum and anti-phase signals cancel. However, the system's response is in terms of the difference between with-control vs. without-control activity. For a linear system, the response is therefore independent of how the system is initialized (e.g., its phase). The dependence upon initial conditions for a non-linear control system is thus distinct from notions of superimposing waves.

## 5. Control concepts III: State-space

For controlling systems with many components and/or non-linearities (both of which apply to the brain), it is also helpful to view the system through a state-space framework. This involves identifying how the system evolves at each time-step as a function of the current state. Each point in state-space corresponds to one pattern of brain activity and a sequence of brain-states (a spatiotemporal pattern) corresponds to a path in state-space (see, e.g., Figure 2C). In the state-space framework, the goal is to find a sequence of inputs that move the system to a desired state (static spatial activity pattern) or along a specified path (spatiotemporal activity pattern). Dynamics determine how the system moves at each step. An optimal control law thus corresponds to a rule that, when followed, eventually drives the system to a desired stopping-place while minimizing costs along the way (e.g., the time taken or the energy used). State-space analysis is particularly central to non-linear systems which generally lack concise, analytic solutions (as opposed to, e.g., Equation 3).

### 5.1. Reachability

Viewing a dynamical system from this perspective (a sequence of paths), a natural question is which portions of the state space (brain activity patterns) may be accessed by an appropriate control? In practice, only limited values of the input signal are allowed [e.g., *u*(*t*) is bounded by safety limits]. The set of allowable inputs are referred to as “admissible”. For a given starting point (*x*_0_), we define the “k-reach set” as the collection of all states that can be reached using an admissible control of length *k*. In other words, the “reach set” consists of all outcomes at time *k* that can be achieved starting at *x*_0_. The *reachable* set consists of all outcomes that can be eventually reached. In other words, reachable sets are the union of reach sets. For a linear system, the initial condition shifts the location of reach-sets but does not alter their shape as the effects of initial conditions and inputs are additive (the “superposition principal”). By contrast, the reach-sets of a non-linear system depend greatly upon the starting position. In the previous simulation of a human MEG model (confined to a limit cycle), the size and orientation of reach-sets is a function of the starting point on the cycle (i.e., the phase; Figure 2C).

**Figure 3:**
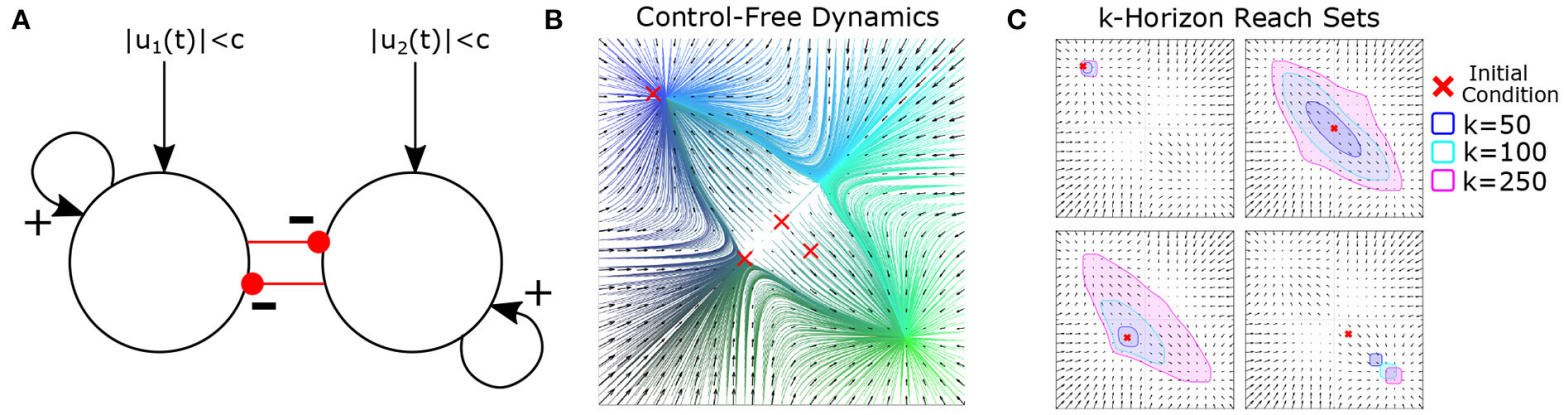
Illustration of state-dependent reachability. **(A)** Schematic of recurrent two-neuron network with self-excitation, reciprocal inhibition, and two independent, bounded, inputs. Bounds on absolute current are denoted by *c* = 0.1. **(B)** Uncontrolled vector field and integral curves (color=initial condition for each curve). **(C)** Approximate reach sets for different initial conditions and time-steps (horizons). Note the strong dependence of initial condition and that reachable sets are larger when starting near a separatrix (e.g., top right) than a fixed point (e.g., top left). Figure and captions reproduced with permission from Singh et al. (2022a). *k* denotes the horizon length (i.e., how long since stimulation started).

We further explore reachability *via* a simulation of two reciprocally inhibiting “neurons” (Figure 3A). We assume that inputs to this system are bounded. In the absence of control, (almost) all initial conditions end at one of two equilibria corresponding to neuron 1 or neuron 2 becoming dominant (Figure 3B). For initial conditions near a (locally) stable equilibrium, the reachable space is small with all solutions eventually getting trapped close to that equilibrium for any admissible controller (Figure 3C top-left and bottom-right) By contrast, initial conditions near an unstable region of state space are able to reach either equilibrium depending upon the controller's input (Figure 3C).

### 5.2. Cognitive interpretation of reachable sets

**Figure 4:**
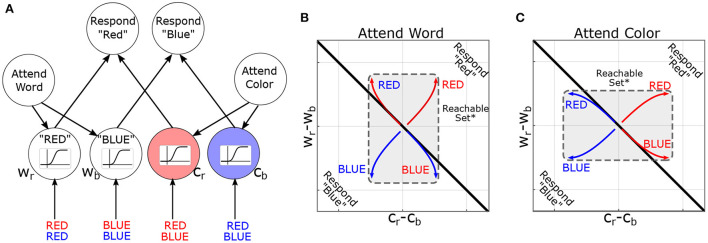
Reachability concept illustrated in the Stroop Task. **(A)** Neural-network model of the Stroop Task (without bias) based on MacLeod and Dunbar (1988) and Cohen et al. (1990), figure adapted with permission from Singh et al. (2022a). Nodes are named according to attribute and value (*C*_*r*_=color-red, *W*_*b*_=word-blue). Sigmoids indicate the activation function: how the activity of a node is converted to its response. **(B)** Simulated trajectories of the model in the “attend word” condition, in response to different word/color combinations. Coordinate axes represent the difference in output between nodes (i.e., *C*_*r*_−*C*_*b*_ is the difference in output between color-red and color-blue nodes). The black line indicates the decision boundary for responding “red” vs. “blue”. *For pedagogy, reachable sets are conceptually illustrated by their convex hull. **(C)** Analogous figure for “attend color”. Note that previous instructions (initial conditions in the model) dilate the reachable set along the corresponding attribute (color or word).

We now explore how control-theoretic methods relate to cognition by modeling the task environment and stimuli as additional inputs to the brain. In this context, the reachable sets describe which patterns of brain activity can be elicited by a class of task stimuli. Too illustrate these concepts, we consider their deployment in a canonical cognitive task: the Stroop task (Stroop, 1935). In the Stroop task, participants are presented with color-words (e.g., the word “BLUE”) and asked to report either the word or the font-color. Thus, each stimulus contains multiple dimensions (word and font-color), and previous instructions dictate which dimension is to be reported. For the present purposes, we are not concerned with asymmetries within the Stroop effect (i.e., the bias toward “word” over “color”). A simple, but influential, model of the task proposes that previous instructions prime which attributes should be attended and thereby “guide” activation in response to future stimuli (Figure 4A). Treating this model as a 6-dimensional dynamical system (2 rule-units + 4 attribute-units), task instructions move the system's initial conditions along the “attend-word” vs. “attend-letter” axis. These initial conditions reshape how subsequent inputs propagate through the network (see Supplementary material for details) by distorting the reachable-set geometry (Figures 4B,C). Previous instructions dilate the reachable set geometry along the cued attribute dimensions: between words (Wr minus Wb) or between colors (Cr minus Cb). This is signified by the central rectangle flipping to be longer on the y-axis for following word reading instructions, but longer on the x-axis following color naming instructions, which signify a larger reachable set on the respective axes. Thus, reachable sets provide a geometric framework for understanding how the initial state of a system constrains its response to subsequent inputs. In this example, the effects are relatively straightforward and easy to understand in conventional terms. However, these effects are less intuitive for larger networks or time-varying inputs. In the next section, we propose leveraging this concept to identify computational properties of brain states and to formulate new neurostimulation objectives.

## 6. Reachability as a control objective

Historically, neurostimulation protocols have been motivated by previous neurophysiology-behavior correlates. This typically takes the form of increasing region-specific brain activity, spectral power, or synchronization between brain areas. Such characterizations are well-documented and concretely-specified. However, there may be some limitations to this framework. Namely, the underlying logic states that because neuro-cognitive process “x” (e.g., working memory maintenance) is associated with signal-feature “y” (e.g., theta-power; Onton et al., 2005); inducing “y” *via* neurostimulation will enhance “x”. However, this inference does not necessarily hold, even when the relationship is causal; i.e., “x” causes “y”. This asymmetry emerges from the difference in spatial scales between the generative neuronal processes and the macroscopic resolution of M/EEG and tES.

As an analogy, consider short-term memory storage in a computer. This process generates a spatially-localized electrical signature: greater current usage by RAM (Random Access Memory). Thus, the relationship between electrical usage and (volatile) memory storage is causal and the former is a necessary condition for the latter. However, uniformly injecting additional current into RAM will not improve its memory capacity (or any other function) as information content is only manifest at microscale (RAM memory cells). Uniformly adding current cannot add new information (other than setting all of the memory cells to “1”). As an aside, we note that the above-referenced limitations concern the ability to enhance cognition beyond what was naturally possible and does not proscribe against conventional methods to restore lost functions. In the computing analogy, for instance, applying a macroscopic current could be useful if the device is unplugged (i.e., by providing a power source) but it can't improve performance beyond the standard operating range (see above arguments).

Likewise, neurophysiological signatures reflect the summation of microscale neuronal activity and many cognitive operations (such as those involving working memory) are inseparable from this scale. For instance, brain regions encoding semantic categories are not easily divided anatomically based upon individual categories (i.e., separating neurons sensitive to the mammal category vs. bird category). Thus, there are significant challenges in determining how macroscale stimulation could enhance higher-cognitive functions in healthy subjects. We suggest that these scales might be bridged through control theory.

Our proposal is founded upon the idea that, for cognitive enhancement, stimulation must be enabling: it improves the brain's ability to find the correct answer to a problem/task, but cannot explicitly move the brain toward the correct answer because (1) the “correct” response is determined by task contexts which are independent of the control law (i.e., the controller cannot perform the task for the subject); and (2) the neural computations involved in higher cognition occur at spatial scales which are inaccessible to the tES input. Instead, we propose to treat the brain as a dynamical system controlled by both tES and the environment, e.g., we write:


(8) $$\begin{array}{l} ẋ=f\left(x,z_{env}\right)+Bu_{stim} \end{array}$$

Treating the environment as a controller-input (*z*_*env*_) we define the reachable-sets under *z*_*env*_ as the values of *x* which, from a given initial condition, can be produced by admissible values of *z*_*env*_. Admissible values of *z* are constrained by known anatomy (the spatial structure of task-evoked activity) and reasonable bounds on magnitude. This reachable set under *z*_*env*_ thus corresponds to how task conditions can affect microscale brain activity. As noted before, the reachable sets depend upon the initial conditions of *x* and the system's dynamics (including any tES feedback). One pathway to cognitive enhancement may be shaping the brain's reachable sets (due to task) by modifying pre-task activity in a proactive manner (setting the brain into an optimal start-point; see Figure 3) or by altering the closed-loop (brain+tES) dynamics. In the next section, we illustrate these ideas in a closed-loop simulation in which macroscale stimulation is used to widen the microscale reachable-sets.

### 6.1. Illustration of concept

**Figure 5:**
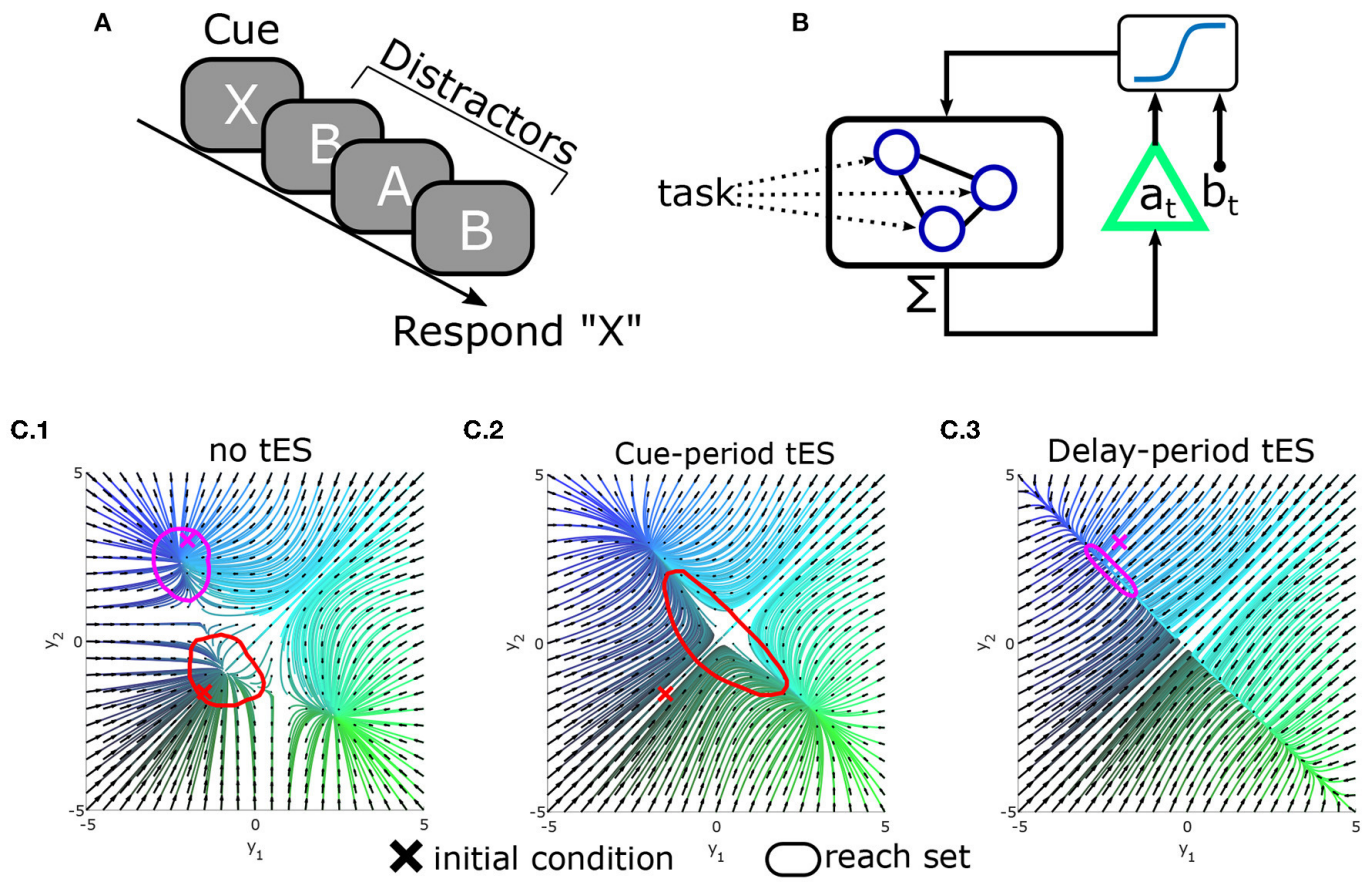
Conceptual demonstration of reachability in a modeled delayed-response task. **(A)** Schematic of the delayed-response task in which three distractors are presented during the delay period. **(B)** Integrated tES+task model. Task stimuli directly interface with microscale neural circuitry: the “neurons” in a leaky-competing-accumulator model. By contrast, the closed-loop controller measures the (macroscale) summed activity (analogous to M/EEG) and stimulates the population as a whole (as opposed to selecting neurons). For simplicity, the controller was modeled as affine-feedback with saturation [i.e., $u_{t}=tanh\left(a\left(\underset{i}{\sum }{\left[x_{t}\right]}_{i}\right)+b\right)$]. The triangle denotes feedback gain/amplification (by *a*), while *b* is an additive bias signal. Separate values of *a, b* are used during the cue vs. delay phases (hence the dependence on time) and *a*_*t*_ = *b*_*t*_ = 0 during the inter-trial period. **(C)** State-space plots of network dynamics (reduced to two neurons for display). Attractors in the model correspond to stable memory representations. **(C.1)** Original dynamics and reach-sets when initialized at baseline (red) or near an attractor (magenta). **(C.2)** Pseudo-optimal settings for *a, b* during the cue-phase increase the reachable space from baseline. **(C.3)** Pseudo-optimal modeled tES settings during delay contract the reachable space so that new inputs (distractors) have less influence.

Clearly the above ideas need to be validated in both simulations and real-world experiments. As a proof-of-concept, we illustrate the potential of tES to alter reachability and improve behavior *in silico* by modeling a continuously performed delayed-response task in which subjects are presented with an item to memorize and report after a delay period with distractors (Figure 5A). To model this process, we use a standard recurrent network model of decision-making with one recurrent unit for each possible item (see Supplementary material; Usher and McClelland, 2001; Wong and Wang, 2006). As each stimulus (cue or distractor) is presented, it excites the corresponding memory unit. Units reciprocally inhibit each-other leading to competition between outcomes. For simplicity, we modeled the behavioral response as the memory unit with greatest activation at the start of the response segment.

In this task, the information processing demands differ between the cue/encoding period and the delay/distractor period. Information accrual is beneficial during the encoding period (when stimuli are goal-relevant) but not during the distractor/delay period. The reachability construct provides a means to manipulate these properties through macroscale interventions while remaining agnostic to the information content itself (i.e., independent of which cue was presented). As a simple demonstration, we explored whether a tES controller, added to this model (Figure 5B), could alter reachability and improve simulated performance. We found that the most effective controller (best task performance) used feedback to increase reachability during the cue/encoding period and decrease reachability during the delay/distractor period (Figure 5C). Thus, the best controller increased reachability for periods in which stimuli were task-relevant (cue/encoding) and decreased reachability during the delay (thereby stabilizing the “memories”). These findings indicate that altering reachability may be one path to improving behavioral accuracy with macroscale stimulation. Ultimately enacting this form of control remains an ongoing endeavor for which technical aspects are still being developed. However, we hope that these ideas demonstrate the relevance of control theory in both the engineering and cognitive aspects of tES design.

## 7. Hurdles to applications in practice

The above ideas demonstrate the ways in which control-theoretic concepts and analyses can benefit brain stimulation paradigms, but clearly several hurdles remain to applying this method in practice. Implementing real-time control often involves several non-trivial steps such as estimating a model of the underlying system, identifying control algorithms, and building the infrastructure to link EEG and tES.

### 7.1. Controller design

The methods and objectives of control theory are broad, but can be largely grouped into closed-loop methods which provide ongoing feedback and open-loop methods in which control is designed and applied independent of ongoing measurements. Closed-loop control (of-varying degrees) is almost always superior but often requires the ability to both measure and manipulate a system simultaneously. This setup is notoriously challenging for non-invasive neurostimulation due to the difficulty of removing stimulation artifact from M/EEG signals.

One path to circumventing artifact contamination is to instead use a hybrid closed-loop approach in which tES is delivered in pulses interleaved with (artifact-free) M/EEG recordings. During active tES, the closed-loop algorithm relies upon simulated values of the expected brain activity at each time step with these values being periodically corrected during the tES-free phases. Several recent EEG+tES studies have already demonstrated the feasibility of an alternating approach by delivering intermittent phase-locked tES (i.e., using intermittently measured EEG to determine the onset of each tACS round; e.g., Reinhart and Nguyen, 2019). A high-level illustration of this idea, combined with the Non-linear Model-Predictive Control (NMPC) algorithm is depicted in Algorithm 1. The NMPC algorithm (see Rawlings, 2000 for an introduction) simplifies the problem of identifying long control sequences by instead only optimizing over short moving horizons into the future at each time-step. This approach thus involves solving a sequence of short optimization problems (one per time-step). The benefit of NMPC is that it efficiently handles constraints (i.e., safety limits on tES current) and does not require concurrent measurements (unlike traditional feedback), while still enabling the controller to adapt when new information is acquired.

**Algorithm 1:** Generic k-step NMPC Algorithm with alternating stimulation/recording intervals. *H*_*t*_ indicates either a stimulation or recording period. The control-objective at each time-step is denoted by *Q*. The *j*-step iteration of *f* is denoted *f*^*j*^

$Û\left(k,{\hat{x}}_{t}\right):={\mathrm{argmin}}_{u_{\left[t,t+k-1\right]}}\overset{k}{\underset{j=1}{\sum }}Q_{t+j+1}f^{j}\left({\hat{x}}_{t},u_{t}...u_{t+j}\right)$
**if** *H*_*t*_=Stim **then**
$û\leftarrow Û\left(k,{\hat{x}}_{t}\right)$
Administer û_*t*_
${\hat{x}}_{t+1}\leftarrow f\left({\hat{x}}_{t},{û}_{t}\right)$
**else**
Record *y*_*t*_
${\hat{x}}_{t}\leftarrow$ State Update given $\left(y_{t},{\hat{x}}_{t-1}\right)$ (e.g., Kalman Filter)
**end if**

### 7.2. System identification

An additional challenge for control relates to obtaining sufficiently accurate models of the brain activity patterns observed in M/EEG (referred to as “system identification” in control-terminology). This step is non-trivial and an area of active research. Because the models must forecast future brain activity, they will almost certainly need to be estimated from timeseries data as opposed to, say, functional/structural connectivity (Honey et al., 2007, 2009). There are several generic approaches for system identification including non-linear autoregressive models, volterra-kernels, and neural networks (Hagan et al., 2002). Additionally, some system identification methods have been specifically developed for neuroscience like Dynamic Causal Modeling (Friston et al., 2003; Kiebel et al., 2008). Most recently, we have presented a new, scalable identification algorithm for large brain models, that we term MINDy (Mesoscale Individualized NeuroDynamic Modeling; Singh et al., 2020, 2022b). Critically, MINDy models are estimated using single-subject M/EEG data based upon the Kalman Filter (Singh et al., 2022b). However, it is clear that there is also significant impetus to improve data-driven model estimation, particularly given the unique challenges (e.g., volume conduction) of M/EEG data.

## 8. Conclusion

In this note, we have advocated for control-theory as a useful framework for the design and analysis of combined neuroimaging+tES experiments. This process can take multiple forms, from using control-theoretic principals to understand previous observations (e.g., frequency selectivity) to optimizing tES using quantitative brain models. We have also identified a mismatch between spatial scales (microscale computation vs. macroscale tES) as a clear hurdle in directly linking neural computation, task conditions/stimuli, and macroscopic fields in a coherent optimization problem. We proposed that control-theoretic measures such as reachability may be able to bridge these scales by shaping the response properties of microscale neurocircuitry without ever accessing or measuring individual circuit components (which are inaccessible). The full integration of closed-loop control and reachability-optimization with tES is surely a multi-step endeavor. However, we hope that, at a minimum, readers will be motivated to further explore control-theory and neuroengineering (see e.g., Schiff, 2011 for an introduction), given the promise of such approaches within this domain.

## Data availability statement

The original contributions presented in the study are included in the article/Supplementary material, further inquiries can be directed to the corresponding author/s.

## Author contributions

MS developed the theory, designed the simulations, and wrote the manuscript. MC, TB, and SC contributed to theory development and edited the manuscript. All authors contributed to the article and approved the submitted version.

## Funding

MS was funded by NSF-DGE-1143954 from the US National Science Foundation, the McDonnell Center for Systems Neuroscience and NIH T32 DA007261-29 from the National Institute on Drug Addiction. Portions of this work were supported by NSF 1653589 and NSF 1835209 (SC), from the US National Science Foundation and NIMH Administrative Supplement MH066078-15S1 (TB).

## Conflict of interest

The authors declare that the research was conducted in the absence of any commercial or financial relationships that could be construed as a potential conflict of interest.

## Publisher's note

All claims expressed in this article are solely those of the authors and do not necessarily represent those of their affiliated organizations, or those of the publisher, the editors and the reviewers. Any product that may be evaluated in this article, or claim that may be made by its manufacturer, is not guaranteed or endorsed by the publisher.
