# Supplementary material for: Control-theoretic integration of stimulation and electrophysiology for cognitive enhancement
Source: Front Neuroimaging. 2022 Nov 18;1:982288. doi: 10.3389/fnimg.2022.982288 (PMC10406304; doi:10.3389/fnimg.2022.982288)
Supplement: Supplementary file 1 [file Data_Sheet_1.pdf]

Supplemental Information for: Control-theoretic Integration of Stimulation and Electrophysiology for Cognitive Enhancement

Matthew F. Singh[a,b,c], Michael W. Cole[b], Todd S. Braver[c], and ShiNung Ching[a]

[a] *Electrical and Systems Engineering, Washington University in St. Louis, St. Louis, 63130, MO, USA*
[b] *Center for Molecular and Behavioral Neuroscience, Rutgers University, Newark, 07102, NJ, USA*
[c] *Psychological and Brain Science, Washington University in St. Louis, St. Louis, 63130, MO, USA*

1. Simulation of Data-Driven Models

In Fig. 2C, we illustrate the reach-sets generated around a limit-cycle. For visualization, we projected data onto the three largest (left) singular vectors of the combined linearized controllability matrix for the three initial conditions. For the T-length orbit $\omega = \omega_0 ....\omega_T$, the first-order approximation is given by the time-varying linearization using deviation variable $[\delta x]$:

$$[\delta x]_{t+1} = F'(\omega_t)[\delta x]_t + Bu_t \tag{1}$$

$$x_t = \omega_t + [\delta x]_t \tag{2}$$

with $\delta_0 = x_0 - \omega_0$ (zero in our case since we started on the limit-cycle). The corresponding controllability matrix (starting from $t_0 = 0$) is given by:

$$C_{t+1} = \Big[B \,\Big|\, F'(\omega_t)B \,\Big|\, F'(\omega_t)F'(\omega_{t-1})B \,\Big|\, ... \,\Big[\prod_{i=1}^{t} F'(\omega_i)\Big]B\Big]. \tag{3}$$

For visualization, we projected data onto the first three singular vectors of the controllability matrix after it was concatenated across initial conditions. Similarly, for visualization, we displayed the linearized (ellipsoidal) reachable sets under the L_2 constraint: $||u|| \leq 4$. These corresponded well with numerically-approximated nonlinear reachable sets for the short-horizons displayed (1/3 period). Details on the models and their estimation is provided in [1]. Note that the size and orientation of reach sets in this first-order expansion still display dependency on the initial values of ω_0 (see Fig. **??**C). As such, these dynamics should not be confused with those of a linear-time-invariant system (in which initial conditions only translate reach sets).

2. Parameterization of Illustrative Models

Simulations of human brain dynamics used two-population models estimated ([1]) from Human Connectome Project MEG data ([2]). Figures illustrate a single subject. Models are of the form:

$$p_{t+1} = W_p \psi_p(p_t) - \beta_p \odot \psi_r(r_t) + \tau_p \odot p_t \tag{4}$$

$$r_{t+1} = W_r \psi_p(p_t) - \beta_p \odot \psi_r(r_t) + \tau_r \odot r_t \tag{5}$$

in which p_t and r_t correspond to pyramidal and inhibitory activity, respectively. The function ψ is the parameterized sigmoid: $\psi(x) = tanh(s \otimes x + v)$.

The network of two reciprocally inhibiting neurons was simulated using the standard recurrent neural-network formulation:

$$\dot{x}_1 = w\psi(x_1) - \beta\psi(x_2) - x_1/\tau + c \tag{6}$$

$$\dot{x}_2 = w\psi(x_2) - \beta\psi(x_1) - x_2/\tau + c \tag{7}$$

with parameters w=1.25, β=.4, τ=4, c=-.4 (this paramaterization is purely illustrative). The activation function is given by a sigmoidal nonlinearity: $\psi(x) := 1/(1 + exp(-[1.5x - .1]))$.

2.1. Memory Task Simulation

The memory task was modeled as one cue followed by three distractor stimuli. All stimuli were presented for 30 time-steps with 40 time-steps in between stimuli. Responses (winner-take-all) were calculated 40 time-steps after the final distractor. The intertrial interval was 50 time steps. The task was simulated as a sequence of 6 trials/block (continuous simulation) with 2000 blocks simulated in parallel. Cues and distractors were independently drawn from a set of three possible stimuli.Neural activity during the memory task was modeled as a nonlinear, leaky, competing network [3] of three neurons ($i = 1, 2, 3$), corresponding to the three possible cue stimuli.

$$x_i(t+1) = x_i(t) + \Delta_t\Big[w\psi_i - \beta\sum_{j\neq i}\psi_j - \frac{x_i(t)}{\tau} + c + I_t + \eta_t + u_t\Big] \tag{8}$$

with ψ_i short-hand for $\psi(x_i(t))$. This discrete-time system was integrated with time step $\Delta_t = .25$ and $\tau = 3$. All other variables (w, β, c, ψ) were the same as the previous example above. The endogenous noise term η_t followed a first-order autoregressive process (similar to [4]):

$$\eta_{t+1} = \eta_t - \Delta_t\frac{\eta_t}{\tau_n} + \sqrt{\frac{\Delta_t}{\tau_n}}\epsilon_t \tag{9}$$

with $\tau_n = 2$. The noise generator, $\epsilon_t \in \mathbb{R}^3$ was uncorrelated, independently realized Gaussian noise for each "neuron" with zero mean, and 0.3 standard deviation. All nodes received a base task input $I_t = .025$ whenever a stimulus was present, regardless of cue/delay phase. The node representing that stimulus received additional task input of 0.3 for cues and .009 for distractors. The controller u_t was implemented as:

$$u_t = tanh\Big(a\sum_i x_i(t) + b\Big). \tag{10}$$

Separate values of a, b were estimated for the cue and delay phases based upon fully-crossed grid search (4 dimensions). The controller was silent ($u_t = 0$) for the inter-trial interval. All nodes received base task input ($I_t = .025$) whenever a stimulus was present, irrespective of trial period. The node corresponding to the presented stimulus also received additional input $I_t = 0.3$ for cue stimuli and 0.09 for distractors. During the inter-trial interval, $I_t = -0.6$ for all nodes.

Acknowledgments

MS was funded by NSF-DGE-1143954 from the US National Science Foundation, the McDonnell Center for Systems Neuroscience and NIH T32 DA007261-29 from the National Institute on Drug Addiction. Portions of this work were supported by NSF 1653589 and NSF 1835209 (SC), from the US National Science Foundation and NIMH Administrative Supplement MH066078-15S1 (TB).

References

[1] M. F. Singh, C. Wang, M. Cole, T. Braver, S. Ching, Efficient identification for modeling high-dimensional brain dynamics, American Control Conference (ACC) 2022 (2022).

[2] L. J. Larson-Prior, R. Oostenveld, S. Della Penna, G. Michalareas, F. Prior, A. Babajani-Feremi, J.-M. Schoffelen, L. Marzetti, F. De Pasquale, F. Di Pompeo, et al., Adding dynamics to the human connectome project with meg, Neuroimage 80 (2013) 190–201.

[3] M. Usher, J. L. McClelland, The time course of perceptual choice: the leaky, competing accumulator model., Psychological review 108 (3) (2001) 550.

[4] K.-F. Wong, X.-J. Wang, A recurrent network mechanism of time integration in perceptual decisions, Journal of Neuroscience 26 (4) (2006) 1314–1328.
